# Neuroglobin: A Novel Player in the Oxidative Stress Response of Cancer Cells

**DOI:** 10.1155/2019/6315034

**Published:** 2019-07-01

**Authors:** Marco Fiocchetti, Virginia Solar Fernandez, Emiliano Montalesi, Maria Marino

**Affiliations:** Department of Science, University Roma Tre, Viale Guglielmo Marconi 446, I-00146 Roma, Italy

## Abstract

Reactive oxygen species (ROS) result from intracellular aerobic metabolism and/or extracellular stimuli. Although endogenous antioxidant systems exquisitely balance ROS production, an excess of ROS production, commonly found in diverse human degenerative pathologies including cancer, gives rise to the oxidative stress. Increased oxidative stress in cancer is related to the sustained proliferation and metabolism of cancer cells. However, cancer cells show an intrinsic higher antioxidant capacity with respect to the normal counterpart as well as an ability to cope with oxidative stress-induced cell death by establishing mechanisms of adaptation, which define a selective advantage against the adverse oxidative stress environment. The identification of survival factors and adaptive pathways, set up by cancer cells against oxidative stress, provides multiple targets for the therapeutic intervention against cancer. Neuroglobin (NGB), a globin primarily described in neurons as an oxidative stress sensor and cytoprotective factor against redox imbalance, has been recently recognized as a novel tumor-associated protein. In this review, the involvement of NGB in the cancer cell adaptation and resistance to oxidative stress will be discussed highlighting the globin role in the regulation of both the stress-induced apoptotic pathway and antioxidant systems activated by cancer cells.

## 1. Introduction

Reactive oxygen species (ROS), including superoxide anion (O_2_^−^), hydrogen peroxide (H_2_O_2_), and hydroxyl radical (OH^·^), are abundant products of aerobic metabolism, and their levels set up the intracellular redox state [1]. However, excessive intracellular ROS levels, not balanced by endogenous antioxidant molecules (*e.g.*, glutathione), could lead to membrane lipid, protein, and DNA damage [1, 2] with the consequent cell death. Thus, the balance between prooxidant and antioxidant compounds influences the cell fate [1–3]. Aside from such a classical view of the intracellular ROS role, in the last two decades, mounting evidence sustained that low concentrations of ROS could promote cell growth and differentiation by regulating the activation status of enzymes, triggering signal transduction pathways as well as gene expression [1, 2].

Cancer cells represent a good example of these dual ROS effects. Actually, cancer cells, compared to their normal counterparts, live constantly or occasionally under an oxidative stress condition [3, 4]. Moreover, the increased ROS levels have been double linked with the tumor initiation and progression. Although the exact mechanism is still unclear, ROS represent the main selective pressure, which could induce cancer cell death or activate aberrant adaptation mechanisms involved in the acquisition of almost all the cancer hallmarks, including sustained cell proliferation, immortalization, cell death escape, metastasis, and chemo resistance [3]. These two faces played by ROS in cancer have been translated into two different strategies to develop anticancer agents. Antioxidant molecules have been predicted as a plausible tumor suppressor based on the tumorigenic role of ROS-activated signaling [3, 4]. However, the role of antioxidant deficit in increased cancer ROS generation and the actual anticancer effects of ROS scavenging are still debated [3]. On the other side, different ROS-inducing chemotherapeutic agents have been developed based on the idea that a further induction of intracellular ROS levels could represent a way to kill selectively cancer cells without affecting the normal counterpart [3, 4]. Nonetheless, the cancer cells' adaptation to high levels of ROS and the consequent cancer resistance associated with the oxidative stress encourage the current research to define the survival factors and activated pathways devoted to increase cancer cell tolerability to ROS. Among different proteins that could serve as a compensatory element, here, we discuss the role of Neuroglobin (NGB), a monomeric heme-protein that operates as an oxidative stress biosensor and player in the context of the compensatory/adaptive systems of cancer cells.

## 2. Neuroglobin as a Stress Sensor in the Brain

NGB is a monomeric heme-protein [5, 6] that displays the classical 3/3 *α*-helical sandwich globin structure and is characterized by the presence of a hexa-coordinated heme-Fe atom in both the ferrous and ferric states [7–9]. Although its discovery date is quite recent, NGB shows a very slow evolutionary rate remaining largely unmodified throughout the evolution, suggesting a critical physiological role of the protein [5, 10, 11]. Due to its belonging to the globin superfamily, initially, it was assumed that NGB might play a role as an intracellular O_2_ carrier [6, 12]. Although such a role could be possible in the retinal cells, where very high NGB concentration (100-200 *μ*M) occurs ([13] and literature cited therein), the relatively low concentration (≤1 *μ*M) found in the other brain area ruled out on the function of NGB as an O_2_ supply [5, 10, 11, 14]. Nonetheless, in the last two decades, the growing interest about NGB is raised from evidence that sustain a cytoprotective function of highly expressed globin in a wide range of neurological disorders and neuronal stressing conditions [5, 15]. In this context, several data have defined the close relationship between NGB and oxidative stress in the brain in terms of NGB ability to preserve cell survival of neuron and astrocytes, *in vitro* and *in vivo*, in the presence of high levels of ROS [16–23]. In addition, increased levels of NGB due to ectopic overexpression protect cultured neurons against hypoxia, oxygen-glucose deprivation (OGD), and neurotoxic challenges induced by sodium arsenite (NaAsO_2_) and *β*-amyloid toxicity, which are directly or indirectly linked to intracellular ROS production [24–27].

In this regard, different hypotheses have been put forward and experimentally tested to define the protective mechanisms of the globin [5, 11, 13]. In particular, it has been proposed that NGB may act as a ROS/RNS scavenger to counteract the increased levels of oxidative stress [5, 28–31]. Nonetheless, the discovery of NGB-interacting proteins and the identification of specific subcellular expression profile (cytosol, mitochondria, nuclei) of the globin have widened the cellular functions in which NGB is involved. The NGB direct impact on the apoptotic pathway [17, 18, 32, 33], the NGB suppression of ROS production through the interaction with the cytochrome *bc_1_* complex (complex III) in the inner mitochondrial membrane [34], and the modulation of several intracellular signaling pathways devoted to the cell survival (*e.g.*, AKT and G protein) [5, 13, 35–39] have been described.

Intriguingly, diverse stress conditions like hypoxia [40], oxidative stress (H_2_O_2_) [17], oxygen and glucose deprivation [41], and lipopolysaccharide [42] increased NGB expression in neuron-derived cells suggesting a role of NGB as a stress-responsive sensor which transfers the stress condition to the signal transduction pathway [43]. On these findings, in neuron-derived cells exposed to oxidative stress, two different and interconnected functions of NGB have been demonstrated: the cytoprotective role, when an excessive intracellular ROS concentration occurs, and the globin involvement in the activated response to internal and external oxidative stress. Furthermore, it has been demonstrated that NGB expression is enhanced by endogenous factors, including hormones and growth factors. Among different hormones (*e.g.*, vascular endothelial growth factor and erythropoietin) [5, 44–46], 17*β*-estradiol (E2) induces, via estrogen receptor *β* (ER*β*), high levels of NGB primarily involved in the E2-activated antiapoptotic pathway in neuron-derived cells [17, 18]. In particular, the demanding localization of NGB into mitochondria for its antiapoptotic function against oxidative stress has been demonstrated. Indeed, only the E2-induced mitochondrial reallocated NGB interacts with and impairs the cytosolic release of cytochrome *c* (Cyt-*c*) preventing the consequent activation of the apoptotic pathway during H_2_O_2_-induced stress condition [18, 47].

Altogether, such functions result in neuroprotection, and any approach able to upregulate NGB could preserve neurons/astrocytes from stress injury. On the other hand, NGB is now considered as an ubiquitary inducible protein, in which increased levels could guarantee the proper response and the adaptation to stress conditions that represent the main mechanisms activated by cancer cells to escape from necrosis and apoptosis in the presence of an imbalanced redox state [4, 48].

## 3. Neuroglobin in Cancer

Some evidence demonstrates the expression of human NGB in the nervous system neoplasm [49–52]. NGB expressions have been found higher in the mouse and the human astrocytoma cell line and in human astrocytoma tissues with respect to the normal astrocytes, sustaining a possible role of NGB in the adaptation of astrocytoma to the hypoxic and oxidative stress conditions [50]. Consistently, the analysis of both NGB mRNA and protein levels strongly sustained an upregulation of NGB levels in glioma tissue with respect to the normal counterpart. The correlation between NGB expression and the worse clinic-pathological feature, type/grade of glioma, poor prognosis, and shorter survival overall led to proposing NGB as a prognostic marker for glioma patients [51, 53]. In particular, Hu and colleagues provided different evidence, which supports the direct role of NGB as an antiapoptotic protein in glioma tumors against oxidative stress. On the one side, they proved that NGB overexpression protects U87 glioma cells against cell death induced by 4-hydroxy-2-nonenal (4-HNE), an end-product of the reaction between ROS and polyunsaturated fatty acids, which reflects a high oxidative stress inside cells. In addition, high levels of the globin preserve glioma cells from the excessive ROS accumulation given by the activation of peroxisome proliferator-activated receptor *γ* (PPAR*γ*) which, in turn, negatively regulates NGB expression to render glioma cells more susceptible to oxidative stress-induced cell death. Noteworthy, in such a contest of bidirectional negative cross-talk between NGB and PPAR*γ*, the expression of such a receptor is lowered during cancer progression in parallel with the increase of NGB in high-grade glioma, strongly sustaining that NGB exerts a critical antiapoptotic function in glioma mainly by protecting cells exposed to accumulating oxidative pressure [54]. Mechanically, it has been proposed that NGB could favor glioma progression and a malignant phenotype by preserving cancer cells from apoptosis against oxidative pressure through the direct regulation of the antiapoptotic PI3K/AKT pathway [53, 54].

Similarly, in other independent studies, the analysis of NGB in matched normal and cancer tissues has demonstrated the enhanced expression of NGB in primary tumors and cancer cell line of brain and nonbrain origins and its positive correlation with a marker of hypoxia conditions [55]. In addition, Oleksiewicz and colleagues confirmed the overexpression of NGB in non-small cell lung cancer (NSCLC) specimens with respect to their matched normal tissue and in a panel of cell lines derived by lung cancer suggesting a NGB procancerous function even in extra-nervous tumors [56].

**Figure 1:**
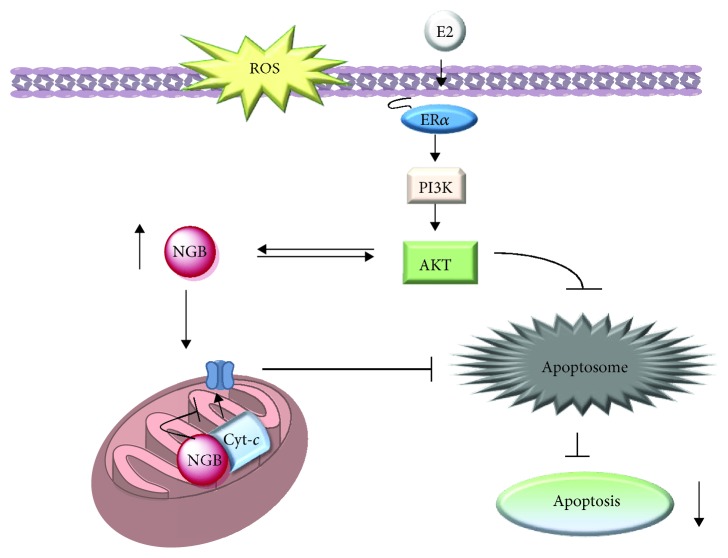
Schematic model of the E2 intracellular activated pathway impacting on NGB expression levels/intracellular localization and the related antiapoptotic role of both mitochondrial NGB and AKT which appears to be double linked with NGB function. E2: 17*β*-estradiol; ER*α*: estrogen receptor *α*; PI3K: phosphatidylinositol 3 kinase, Cyt-*c*: cytochrome *c*. For further detail, see the text.

In line with the above reported results, we identified NGB as a 17*β*-estradiol- (E2-) inducible protein and key mediator of the hormone functions in various estrogen-responsive extra-nervous cancer cell lines [57–59]. Intriguingly, E2 upregulates NGB in diverse breast cancer cell lines (MCF-7, T47D, and ZR-75-1) expressing just the subtype *α* of the estrogen receptor (ER*α*), but not in the ER*α*-devoid MDA-MB-231 cell line [58, 59]. Although ERs are ligand-activated transcription factors, the *NGB* gene promoter does not contain any estrogen response element; thus, multiple and synergic cellular mechanisms underline the E2-induced NGB expression. Physiological E2 concentrations, in the presence of ER*α*, rapidly activate the pathway PI3K/AKT, which in turn prevents proteasome and lysosomal NGB degradation, and enhance *NGB* gene transcription via the phosphorylation of the nuclear transcription factor CREBP [58, 59]. Moreover, the persistent (24 h) AKT activation is necessary to reallocate NGB to the mitochondria (Figure 1) [58, 59]. Intracellular (*e.g.*, ROS) and extracellular stresses that cause the loss of mitochondrial membrane integrity and the release of Cyt-*c* from cardiolipin represent the main trigger event, which commits the cell to death [60]. Indeed, once released, Cyt-*c* binds to the apoptosis protease activation factor (APAF-1) to form the apoptosome that, in turn, activates effector caspases leading to apoptotic cell death [60]. Mitochondrial NGB localization, induced by E2, binds to free Cyt-*c* avoiding its release in the cytosol and the consequent apoptosome formation [18, 61]. Thus, NGB upregulation is one of the critical mechanisms triggered by the E2/ER*α* complex to protect breast cancer cells against oxidative stress by preventing, at mitochondrial levels, the triggering of the apoptotic cascade (Figure 1) [58, 59]. A similar E2-induced antiapoptotic function has also been reported in the hepatoma cell HepG2 [59] in contrast with the antiproliferative and tumor-suppressor function of the overexpressed NGB reported by other authors in these cells [39].

Overall, beyond the contradictory evidence about the expression of NGB in tumor cells and tissues [39, 62], mostly affected by experimental procedure, new perspectives regarding a possible role of the protein as a part of the defense mechanism against oxidative stress in cancer occurred.

## 4. Neuroglobin and Oxidative Stress Signaling in Cancer

A large number of positive and negative regulator systems affect the balance between oxidative stress and antioxidant capability, many of these systems are significantly modified in cancer cells leading to an aberrant regulation of redox homeostasis [3, 4]. Persistent ROS exposure in cancer cells may lead to cell adaptation via the abnormal activation of different redox-sensitive transcription factors including nuclear factor-*κ*B (NF-*κ*B), c-Jun, hypoxia-inducible factor-1 (HIF-1), and the nuclear factor erythroid 2-related factor 2 (NRF-2) whose functions are largely involved in the positive expression of different antioxidant enzymes (*e.g.*, SOD, catalase, and GSH antioxidant systems) [3, 4]. Aside from these redox-sensitive transcription factors, the forkhead box O (FOXO) family of transcription factors and p53 also have a major role in the regulation of antioxidant system expression. Both FOXO and p53 results are strongly modified during the onset and progression of cancer [1, 3].

In the classical stress response pathway consisting of sensors, signal transducers, and effectors, different proteins are regulated through redox-mediated mechanisms behaving, effectively, as an oxidative stress sensor [3]. ROS can change both the levels of proteins acting on their stability or expression and their functions through a direct regulation of structural conformation and reactivity by redox reaction on cysteine residues or an indirect posttranslational modification tightly regulated by redox-sensitive signaling proteins [3]. Altogether, upstream stress sensing and transducer systems, which are deeply modified during unbalanced and aberrant conditions, could represent good targets for the therapeutic approach direct to impact on cancer oxidative stress regulation.

Human NGB contains three cysteine residues; those at positions CD7 (cysteine 46) and D5 (cysteine 55) are sufficiently close to form a disulfide bond. This defines a redox-dependent conformational transition in NGB between a structure with intramolecular disulfide bond, oxidized form, and a disulfide-free NGB form in reduced condition [63–65]. Overall, NGB could sense the intracellular status, in terms of redox state or activation/inactivation of signaling cascade, by changing its three-dimensional structure, which mainly affects the protein affinity with an endogenous gaseous ligand, including oxygen [63–65], and, reasonably, could regulate NGB functional properties including its interaction with proteins (interactome) and intracellular localization as well.

In neuron-derived cells, Watanabe and colleagues demonstrated that oxidative stress imposes a large tertiary modification of the NGB fold allowing its interaction with flotillin-1 at the plasma membrane and favoring its inhibitory function on G*α_i_* protein and the consequent oxidative stress-induced apoptosis, thus acting as an oxidative stress sensor able to impact on cellular response [43]. A similar oxidative stress-sensing activity has also been proposed in malignant tumor cells. In hepatoma cells, evidence suggests a role of NGB as on oxygen/ROS sensor, where it could act by coupling oxygen/ROS signals with a signal cascade, in particular, suppressing the Raf/MEK/ERK pathway via a regulatory machinery, which may involve other NGB-interacting proteins [39].

**Figure 2:**
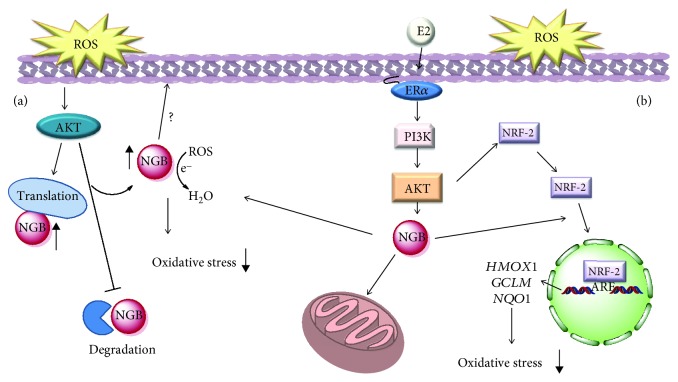
(a) Schematic model of ROS-activated signaling involved in the rapid modulation of NGB levels, its localization, and function on the redox balance outside mitochondria. (b) Schematic model of the E2 intracellular-activated pathway impacting on NGB expression, localization, and the NRF-2 pathway describing how NGB affects the E2-dependent activation of the antioxidant NRF-2 system. E2: 17*β*-estradiol; ER*α*: estrogen receptor *α*; PI3K: phosphatidylinositol 3 kinase. For further detail, see the text.

In this context, we recently confirmed NGB as a stress-inducible protein in breast cancer cells, where it acts as a sensing and compensatory protein activated in response to oxidative stress [59, 66]. As reported above, oxidative stress might affect the activity of sensor proteins by changing their levels via different ways. In our study, we demonstrated that oxidative stress mainly increases NGB levels by acting, like E2, through the inhibition of lysosomal protein degradation and the increase of the protein translation rate [66]. In particular, in breast cancer cells, our evidence demonstrated the transient activation of the PI3K/AKT signaling cascade by oxidative stress which culminates in NGB upregulation and in its localization mainly at the cytosolic compartment, where it could act as a direct ROS scavenger, behaving as a first barrier to the increased ROS levels (Figure 2) [58].

Conversely to the mitochondrial-gathered NGB induced by E2 (see the previous section), the oxidative stress-dependent increase in cytosolic NGB content does not correlate with a direct antiapoptotic function, opening new perspectives in the NGB function during an imbalanced stress condition depending on its intracellular localization [58].

Among the others, NRF-2 is the main regulator of the intracellular defense mechanism against oxidative stress controlling the transcription of ARE (antioxidant-responsive elements) containing genes encoding for proteins (*e.g.*, *glutamate-cysteine ligase modifier* (*GCLM*), *heme oxygenase (decycling) 1* (*HMOX1*), and *NADPH:quinone oxidoreductase 1* (*NQO1*)) involved in cell detoxification of reactive species [67, 68]. Under the resting condition, NRF-2 is targeted to the proteasome degradation by the binding with the oxidative sensor Kelch-like ECH-associated protein 1 (KEAP-1). KEAP-1 interaction with NRF-2 is mainly modulated by the intracellular redox state; indeed, high levels of oxidative stress oxidize KEAP-1 cysteine residues leading to the dissociation of its complex with NRF-2, which accumulates in cells and translocates to the nucleus where it induces the transcription of antioxidant genes [1, 69].

Some evidence addresses NRF-2 as a tumor suppressor function in normal and premalignant cells, according to the role of oxidative stress on cancer onset [4, 70]. Contrarily, a constitutive stabilization of NRF-2 and the consequent high levels of antioxidant enzymes are often found in malignant tumor cells and tissue, sustaining that an increased detoxifying intracellular system confers an advantage for the cancer progression and adaptation to microenvironment stressing conditions [1, 69]. In solid tumors, including those of the lung and liver, somatic missense mutations of the *KEAP-1* gene, which result in a mutant KEAP-1 protein unable to mediate the NRF-2 degradation, have been found. Consistently, mutations in the NRF-2 gene observed in cancer and linked to a constitutive hyperactivation of the transcriptional function of the protein are totally related to the critical site for the formation of NRF-2 and KEAP-1 complex ([69] and literature cited therein).

Our latest results support a critical role of NGB as cytosolic signals intermediate in breast cancer cell stress response, taking part in the E2-dependent activation of the NRF-2 pathway and potentiation of the antioxidant system. Indeed, although the E2-dependent increase in the NRF-2 protein levels was not affected in NGB knockout MCF-7, the positive modulation of antioxidant genes under the direct control of the NRF-2 pathway (*NQO1*, *HMOX1*, and *GCLM*) was lost. As a consequence, obtained data strongly sustain a key function of NGB in the E2 and NRF-2-dependent high antioxidant capacity of estrogen-responsive breast cancer cells, and they suggest a possible role of the globin in the cytosolic signaling pathway involved in the NRF-2 nuclear translocation and transcriptional activity (Figure 2) [71].

The current understanding of NGB function, in particular regarding its involvement in oxidative stress response, is mainly focalized on the intracellular content of NGB and its subcompartmentalization. However, latest reported data propose an effect of subnanomolar concentration of extracellular NGB on the cellular function regulation, in terms of cytoprotection and modulation of antioxidant response [72]. In particular, authors found out that astrocyte cell treatment with extracellular NGB significantly reduced the H_2_O_2_ cell death by restoring the cell antioxidant systems, as demonstrated by the increase in SOD and catalase enzyme activity and transcription. The AKT phosphorylation appears to be the critical signaling pathway activated by exogenous NGB [52, 72, 73]. In addition, active AKT regulates the NGB expression and globin delivery inside cells in a time-dependent manner [58, 59]. Therefore, the story of the double cross link between AKT and NGB adds another piece of information widening the possible interaction between the globin and the PI3K/AKT signaling pathway. As a whole, such results open the fascinating possibility that, in cancer tissue, like in the brain, the NGB function in the cell adaptation response to oxidative stress might not be restricted to the intracellular environment but also spread to the extracellular microenvironemnt. In this context, the overproduced NGB in cancer cells by endogenous modulators (*e.g.*, E2) or stress conditions (*e.g.*, H_2_O_2_) may be, under proper circumstances, delivered outside the cells, participating in a cellular response to external stimuli which appear not confined to the cell itself but rather extended to the cell-microenvironment interface. However, further studies will be needed to properly support such a hypothesis and better understand the possible mechanism of action of NGB outside the cells.

## 5. Conclusion and Perspectives

The balance between ROS and the antioxidant-activated systems set up the levels of intracellular stress, and the fine regulation of this equilibrium is demanding for the correct function and survival of cells [4, 74]. At cellular levels, ROS-scavenging largely relies on the activation of enzymatic processes such as superoxide dismutase (SOD), glutathione peroxidase, catalase, glutaredoxin, and thioredoxin which, overall, constitute the antioxidant defense mechanism of the cells [3, 74]. When the intracellular antioxidant capability is overwhelmed by ROS, cytoprotective mechanisms are activated to impair, when possible, cell death due to the oxidative damage [74].

Given the aberrant ROS concentration, which commonly occurs in tumor progression, cancer cells establish several adaptive mechanisms, including antioxidant responses or activation of prosurvival pathways (*e.g.*, Bcl-2 and AKT), to cope with such stress and survive [3, 4]. Therefore, a greater understanding of pathways devoted to increase the threshold of ROS cellular tolerability appears to be demanding to overcome oxidative stress-associated cancer resistance and to improve the efficacy of therapeutic intervention in selectively killing cancer cells.

Since its discovery in 2000, NGB has been considered a specific globin expressed in the nervous system [6, 75, 76] where its neuroprotective function against different neurological disorders has been demonstrated in both *in vivo* and *in vitro* studies [17, 21, 22, 40, 77, 78]. Aside from this, different evidence sustain a role of NGB as a novel tumor-associated protein [39, 55, 56, 59]. Although the exact role of NGB as a cancer promotor or oncosuppressor is still debated [39, 55, 56, 59], the discovery of higher NGB expression in tumor tissues with respect to the normal counterpart in the brain and extra-nervous malignancies [55, 56] and the positive correlation between the globin expression and glioma tumor grade [54] lead to sustaining a critical NGB function in cancer progression. Consistently, NGB is directly correlated, at different levels, with the cancer cells' adaptation to the increased oxidative stress, which characterized the tumor microenvironment. On the one side, the globin interferes with the apoptotic cascade activated by ROS [53, 58, 59]; on the other side, it participates in the stress response to the detrimental redox imbalance [54, 55, 71].

In the last decade, increasing efforts have been made to define the molecular mechanisms behind the neuroprotective effects of NGB, which, overall, might also occur in tumors. Some of the proposed mechanisms, including O_2_ carrier and ROS scavenger functions, have arisen looking at the typical globin structure of the protein [11, 31, 64, 79]. Furthermore, the involvement of NGB in regulating membrane/cytosolic transduction pathways devoted to increase cell survival [43, 53, 73, 80] and/or mitochondrial functionality [18, 21, 58, 59] has been demonstrated both in neurons/glial cells and cancer cells. The high reactivity of NGB protein in response to change in the intracellular redox state [5], its differential intracellular localization (*e.g.*, cytosol and mitochondria) depending on extracellular stimuli [58, 66], and the large spectrum of NGB-interacting proteins [13] sustain the idea that NGB functions may be finely regulated by the cross-action of all of these events. This intriguing vision is supported by evidence in cancer cells indicating that the antiapoptotic role of NGB against oxidative stress required the protein mitochondrial localization [58, 59], and it is promoted by the change in reactivity and protein interactions (*e.g.*, Cyt-*c*) induced by oxidative stress itself [57]. In parallel, cytosolic NGB directly participates in the antioxidant system established by cancer cells to cope with enhanced ROS accumulation, so that one might predict that different pools of intracellular NGB may cooperate through different mechanisms to enhance cancer cell survival and promote cell adaptations to the stressful tumor microenvironment. In this regard, any stimulus able to change the NGB levels, intracellular localization, and reactivity in order to withdraw NGB prooncogenic functions might be promising to increase cancer cell susceptibility to oxidative stress-induced cell death.

Globins (i.e., myoglobin (MB), hemoglobin (HB), cytoglobin (CYGB), and NGB) are present in all kingdoms of living organisms where they display a variety of functions, including the O_2_ sensing, transport, and storage; the synthesis and scavenging of RONS; and heme-based catalysis [81]. However, many studies demonstrated various other and additional roles of globins including the regulation of cancer progression. The rather low expression levels of these globins in tumor tissue seems to argue against a contribution to tumor oxygenation [62]. On the other hand, the reported functions of MB and CYGB in breast and lung tumors agree with the proposed role as a tumor suppressor for these globins [82, 83]. Contrarily, NGB is the unique globin identified as a critical player in cancer cell adaptations and resistance to detrimental oxidative stress conditions. Although further studies are needed to understand the complex regulating mechanisms of NGB functions, the NGB role in cancer could represent the new target for anticancer therapeutic interventions.
